# TRPS1 expression in breast angiosarcoma

**DOI:** 10.1007/s00428-024-03852-2

**Published:** 2024-06-20

**Authors:** Tamás Pancsa, Boglárka Pósfai, Anna Schubert, Szintia Almási, Eszter Papp, Yi-Che Chang Chien, Endre Kálmán, Kristóf Attila Kovács, Janina Kulka, Linda Varga, Gábor Cserni, Levente Kuthi

**Affiliations:** 1 Department of Pathology, Albert Szent-Györgyi Medical School, University of Szeged, Szeged, Hungary; 2 Department of Surgical and Molecular Pathology, Tumor Pathology Center, National Institute of Oncology, Budapest, Hungary; 3 Department of Pathology, Faculty of Medicine, University of Debrecen, Debrecen, Hungary; 4 Department of Pathology, Faculty of Medicine and Clinical Center, University of Pécs, Pécs, Hungary; 5 Department of Pathology, Forensic and Insurance Medicine, Semmelweis University, Budapest, Hungary; 6 Department of Oncotherapy, Albert Szent-Györgyi Medical School, University of Szeged, Szeged, Hungary; 7 Department of Pathology, Bács-Kiskun County Teaching Hospital, Kecskemét, Hungary; 8 Department of Pathology and Experimental Cancer Research, Semmelweis University, Budapest, Hungary

**Keywords:** Angiosarcoma, Breast, TRPS1, Immunohistochemistry

## Abstract

Angiosarcoma (AS) of the breast, a rare mesenchymal neoplasm, exhibits distinct forms based on etiological and genetic features. While cases with typical clinical presentation and morphology allow for a straightforward diagnosis, challenges arise when clinical data are scarce, diagnostic material is limited, or morphological characteristics overlap with other tumors, including undifferentiated carcinomas. The trichorhinophalangeal syndrome protein 1 (TRPS1), once regarded as highly specific for breast carcinomas, now faces doubts regarding its reliability. This study explores TRPS1 expression in breast AS. Our investigation revealed that 60% of AS cases displayed TRPS1 labeling, contrasting with the 40% lacking expression. Scoring by four independent readers established a consensus, designating 12/35 ASs as unequivocally TRPS1-positive. However, uncertainty surrounded nine further cases due to a lack of reader agreement (being substantial as reflected by a kappa value of 0.76). These findings challenge the perceived specificity of TRPS1, shedding light on its presence in a noteworthy proportion of breast ASs. Consequently, the study underscores the importance of a comprehensive approach in evaluating breast ASs and expands the range of entities within the differential diagnosis associated with TRPS1 labeling.

**Supplementary Information:**

The online version contains supplementary material available at 10.1007/s00428-024-03852-2.

## Introduction

Angiosarcoma (AS) of the breast represents a rare mesenchymal neoplasm, constituting a mere 0.2% of all malignant breast tumors [1]. This unique malignancy is characterized by a range of morphologies from lobulated capillary angioma-like areas or cavernous vascular spaces reminiscent of benign angiomas to the presence of highly atypical epithelioid and spindle cells, forming vessels of varying sizes and shapes [2]. Clinically, breast AS manifests in two distinct forms: primary (de novo) and secondary or better known as radiation-associated angiosarcoma (RAAS) [3]. The primary variant originates deep within the breast parenchyma, predominantly affecting younger women, and exhibits no correlation with prior radiotherapy [4]. Conversely, RAAS emerges as an iatrogenic tumor following radiotherapy for breast cancer and involves the skin and later the underlying superficial breast parenchyma, if any [2]. The global incidence of RAAS is on the rise [5]. Importantly, the genetic profiles of these two forms differ significantly, with *MYC* amplification being a distinctive feature of RAAS rarely observed in the primary variant [6]. In general, the prognosis for both forms remains poor [7]. Multiple local recurrences are frequent in both forms, and the common metastatic sites include the lungs, liver, and bones [8]. Primary AS has a slightly better outcome (median disease-free survival 2.8 years) compared to RAAS [4]. It must be noted that literature data on AS survival is limited [9].

The trichorhinophalangeal syndrome protein 1 (TRPS1) is a zinc finger transcription factor belonging to the GATA family, playing a pivotal role in breast carcinoma development [10]. TRPS1 is physiologically also crucial for bone and cartilage tissue development [11], and recent findings have highlighted its expression in synovial sarcomas [12]. In the context of breast sarcomas, TRPS1 expression has been identified in malignant phyllodes tumors but not in liposarcoma or AS [13]. In contrast, breast carcinoma is marked by elevated TRPS1 expression, underscoring its significance in carcinogenesis and tumor cell survival [14]. Notably, TRPS1 staining is observed in both hormone receptor-positive and triple-negative breast cancers, making it an attractive immunohistochemical marker in diagnostic work [15, 16]. Limited data exist regarding TRPS1 positivity in other primary breast neoplasms. This study aims to explore TRPS1 expression in breast AS.

## Materials and methods

### Study population

A total of 38 samples initially diagnosed as breast AS were collected from the archives of the University of Szeged, University of Debrecen, University of Pécs, Semmelweis University, Bács-Kiskun County Teaching Hospital, Szent Margit Hospital, and National Institute of Oncology. The cohort encompassed one bilateral and two recurring AS cases, from 35 patients. A critical final review of all cases was performed by two of the authors (TP and LK). During this review process, three samples were excluded due to inappropriate histological diagnoses. Specifically, one tumor was identified as metaplastic carcinoma, another was reclassified as an atypical vascular lesion (AVL), and the third was characterized as granulation tissue with pseudosarcomatous changes. The refined study cohort comprised 35 AS cases from 32 patients. Essential pathological parameters including size, laterality, dominant growing pattern, dominant tumor cell morphology, presence of tumor cell necrosis, and mitotic count were recorded. The tumor grade was evaluated using the system established by Kuba et al. solely for the primary AS case [17]. Additionally, comprehensive clinical and pathological data related to any prior breast carcinoma (if there was any) were extracted from the records of the participating institutes and hospitals. The time interval between radiotherapy for breast carcinoma and the onset of AS was systematically calculated.

### Immunohistochemistry

All immunohistochemical (IHC) staining procedures were conducted in a single laboratory, specifically the Department of Pathology at the University of Szeged, utilizing the Leica BOND-MAX Fully Automated Staining System from Leica Biosystems (Deer Park, IL). The IHC assays were executed on the most representative formalin-fixed paraffin-embedded block of the tumors. The panel of antibodies employed for immunohistochemistry included CD31, ERG, MYC, and TRPS1 (rabbit polyclonal, 1:250 Invitrogen, Waltham, MA). CD31 (mouse monoclonal, 1:100, Cell Marque, Rocklin, CA), ERG (EP111, rabbit monoclonal, 1:500, Cell Marque, Rocklin, CA), and MYC (EP121, rabbit monoclonal, 1:100, Cell Marque, Rocklin, CA) staining assessments were collaboratively scored by two pathologists (TP and LK) according to the following categorization: negative (absence of staining or < 1% positive tumor cells), focally positive (1–50% of tumor cells positive), and diffusely positive (> 50% of tumor cells positive). TRPS1 evaluation was evaluated independently by four pathologists (TP, SzA, GCs, and LK) and was interpreted as positive if at least 10% of tumor cells exhibited nuclear TRPS1 expression of any intensity. Employing these standardized criteria, we classified the cases as either positive or negative, if there was a unanimous agreement among the readers.

### Statistical analysis

Pearson’s chi-square test was employed to ascertain significant differences among morphological parameters and TRPS1 expression, with significance defined as *p* < 0.05. To measure the inter-rater agreement among the readers for TRPS1, the Fleiss’ kappa statistic was applied. The results were interpreted as follows: slight (0–0.2), fair (0.21–0.4), moderate (0.41–0.6), substantial (0.61–0.8), and almost perfect (> 0.8) agreement. The statistical analyses were performed using the SPSS software (IBM, Armonk, NY) package.

### Statement of ethics

This retrospective non-interventional diagnostic study was conducted with the approval of the Scientific and Research Ethical Committee of Medical Research Council of Hungary (ETT TUKEB, BM/17641–3/2023). An exemption from individual informed consents was part of the approval.

## Results

### Clinical characteristics

Our cohort comprised 34 cases of RAAS and one case of primary AS. The primary AS manifested in a lady with known Milroy syndrome at the age of 33 years. Among the patients with RAAS, the median age was 73.5 years (mean, 71.4 years; range, 45–89 years). One patient presented with metachronous bilateral AS, one patient experienced recurrent tumors, and another patient’s AS produced metastasis to the contralateral breast parenchyma. Surgical resection was performed in 33 cases, while one tumor was identified postmortem, and another was diagnosed through core biopsy sampling. Regarding RAASs, all patients had a history of prior breast carcinoma, classified into the following histological types: 18 invasive carcinomas of no special type, four invasive lobular carcinomas, one ductal carcinoma in situ, one intraductal papillary carcinoma (probably corresponding to encapsulated papillary carcinoma), whereas the histological type remained unknown in eight cases. The median age of patients at the time of breast carcinoma diagnosis was 63 years (mean, 62.5 years; range, 37–83 years). The median time interval between the diagnosis of breast carcinoma and the onset of RAAS was 8 years (mean, 8.9 years; range, 1–23 years). A summary of the principal clinical data and the features of prior breast carcinomas are presented in Table 1.

**Table 1:** The clinicopathological characteristics of the patients studied. AS indicates angiosarcoma; *Gy*, gray (unit); *NST*, carcinoma of no special type; *ND*, no data; *DCIS*, ductal carcinoma in situ; *ILC*, invasive lobular carcinoma; *L*, left; *Ri*, right; *P*, primary; *Re*, relapse; *M*, metastasis

Patient ID	Age (years)	Histological subtype of prior breast carcinoma	Time interval between breast carcinoma and AS (years)	Radiotherapy, full dose in Gy
Primary angiosarcoma				
1	33	-	-	-
Secondary angiosarcoma				
2	79	NST	15	Yes, ND
3	75	NST	7	Yes, 66.4
4	51	NST	6	Yes, 50.4
5	80	NST	9	Yes, 50.4
6	72	DCIS	10	Yes, 50
7	74	NST	16	Yes, 50
8	83	NST	6	Yes, 50.74
9	56	No data	7	Yes, ND
10	69	No data	16	Yes, ND
11	45	NST	8	Yes, 64
12	80	ILC	9	Yes, ND
13	80	NST	9	Yes, 66
14	74	Intraductal papillary carcinoma	10	Yes, 50
15	66	NST	17	Yes, ND
16	81	No data	23	Yes, ND
17	89	NST	6	Yes, ND
18	64	NST	1	Yes, ND
19	64	NST	1	Yes, ND
20	68	ILC	4	Yes, ND
21	65	No data	ND	Yes, ND
22	74	No data	ND	Yes, ND
23	77	NST	5	Yes, ND
24	80	NST	7	Yes, ND
25, L	73	NST	6	Yes, 49
25, R	69	No data	11	Yes, ND
26, P	56	ILC	8	Yes, 54.6
26, Re	57	-	-	-
27, P	64	NST	10	Yes, ND
27, M	57	-	-	Yes, ND
28	78	No data	ND	Yes, ND
29	82	No data	9	Yes, ND
30	78	NST	7	Yes, ND
31	68	ILC	7	Yes, 60
32	71	NST	9	Yes, 50

### Pathological characteristics

#### Radiation-associated AS

**Fig. 1:**
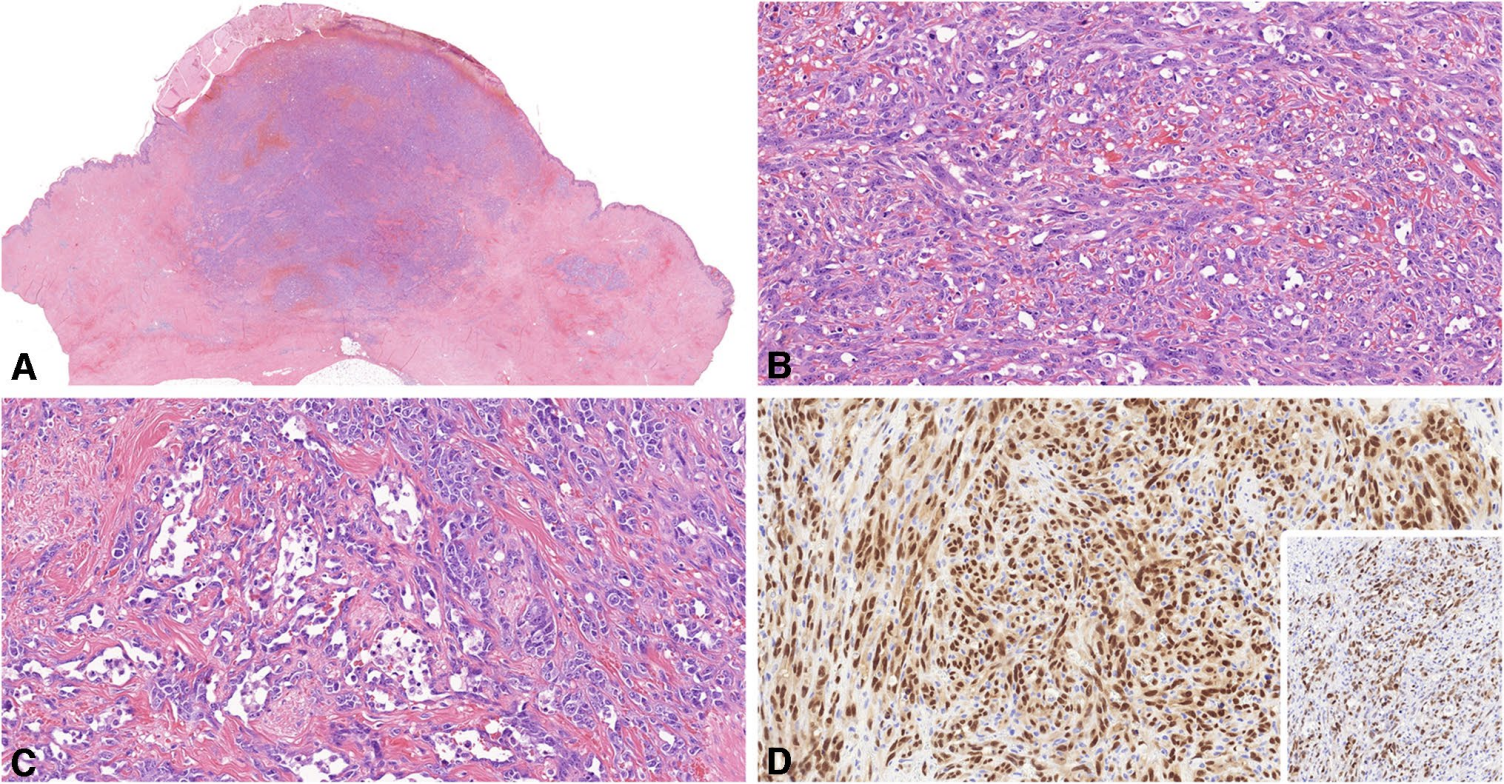
Histological characteristics of a radiation-associated angiosarcoma. **A** At a low-power view, a hypercellular tumor fills the dermis nearly throughout its entire thickness, with evidence of tumor ulceration (HE, × 0.8). **B** The tumor is comprised of both epithelioid and spindle-shaped cells, displaying severe cytological atypia and brisk mitotic activity (HE, × 20). **C** At the tumor’s periphery, asymmetrical vessels covered by atypical endothelial cells are evident (HE, × 20). **D** Immunohistochemical analysis reveals a diffuse ERG labeling in tumor cells, along with a strong and diffuse MYC expression [insert photo] (both × 20)

Among the RAAS cases, 28 tumors originated from a single tumor focus, encompassing a relapsed tumor and a metastasis to the contralateral breast parenchyma. Additionally, six tumors exhibited a multifocal origin. The median size of the tumors was 44 mm (mean, 44.7 mm; range, 5–130 mm). Macroscopically, most tumors displayed a hemorrhagic plaque-like appearance on the skin surface, although advanced cases exhibited infiltration into both the skin and breast parenchyma. Representative images illustrating the gross morphology of the tumors are presented in Supplementary Fig. 1. Twenty tumors developed in the left breast, while 12 emerged on the right side. Histologically, within the tumors examined, 28 displayed a predominant solid growth pattern, while six exhibited a vasoformative architecture. The majority of tumors consisted of pleomorphic tumor cells (*n* = 14), followed by spindle cells (*n* = 12), and epithelioid cells (*n* = 8). Furthermore, tumor cell necrosis was evident in eight cases. A brisk mitotic activity was observed in the majority of cases (mean mitotic count per mm^2^ = 12). All cases exhibited expression of CD31, ERG, and MYC. Representative images depicting the histological features of the tumors are displayed in Fig. 1, and a summary of the pathological parameters along with the immunoprofile of the RAAS cases is provided in Table 2.

**Table 2:** The pathological data of the tumors analyzed. L indicates left; *R*, right; *P*, primary tumor; *Re*, relapse; *M*, metastasis

Patient ID	Size (mm)	Laterality	Multifocal	Dominant growing pattern	Dominant tumor cell morphology	Mitoses per mm^2^	Tumor cell necrosis	CD31	ERG	MYC
Primary angiosarcoma										
1	210	Left	Yes	Vasoformative	Epithelioid	2	No	+ +	+ +	−
Radiation-associated angiosarcoma										
2	32	Right	No	Solid	Epithelioid	25	No	+ +	+ +	+ +
3	5	Left	Yes	Solid	Spindle	16	No	+ +	+ +	+ +
4	12	Right	Yes	Solid	Spindle	3	No	+ +	+ +	+ +
5	55	Left	No	Solid	Pleomorphic	24	No	+ +	+ +	+ +
6	70	Left	Yes	Solid	Spindle	3	No	+ +	+ +	+ +
7	30	Left	No	Solid	Pleomorphic	7	No	+ +	+ +	+ +
8	64	Right	Yes	Solid	Pleomorphic	27	No	+ +	+ +	+
9	100	Right	No	Solid	Pleomorphic	14	Yes	+ +	+ +	+
10	45	Left	No	Solid	Spindle	1	No	+ +	+ +	+ +
11	90	Left	Yes	Solid	Pleomorphic	10	No	+ +	+ +	+ +
12	80	Left	Yes	Solid	Epithelioid	7	No	+ +	+ +	+ +
13	130	Left	No	Solid	Pleomorphic	10	Yes	+ +	+ +	+
14	10	Left	No	Vasoformative	Epithelioid	5	No	+ +	+ +	+ +
15	40	Left	No	Solid	Pleomorphic	20	No	+ +	+ +	+ +
16	50	Right	No	Vasoformative	Epithelioid	3	No	+ +	+ +	+ +
17	70	Right	No	Solid	Spindle	9	No	+ +	+ +	+
18	10	Right	No	Solid	Pleomorphic	8	No	+ +	+ +	+ +
19	5	Right	No	Vasoformative	Epithelioid	1	No	+ +	+ +	+ +
20	11	Left	No	Solid	Spindle	11	No	+ +	+ +	+ +
21	12	Left	No	Solid	Pleomorphic	16	No	+ +	+ +	+ +
22	18	Right	No	Solid	Pleomorphic	38	No	+ +	+ +	+ +
23	10	Left	No	Vasoformative	Epithelioid	5	No	+ +	+ +	+ +
24	22	Right	No	Vasoformative	Epithelioid	18	No	+ +	+ +	+
25, L	60	Left	No	Solid	Spindle	2	No	+ +	+ +	+
25, R	45	Right	No	Solid	Pleomorphic	10	No	+ +	+ +	+
26, P	30	Left	No	Vasoformative	Spindle	4	Yes	+ +	+ +	+ +
26, Re	21	Left	No	Solid	Spindle	4	No	+ +	+ +	+ +
27, P	110	Left	No	Solid	Pleomorphic	12	No	+ +	+ +	+ +
27, M	110	Right	No	Solid	Pleomorphic	27	Yes	+ +	+ +	+ +
28	60	Left	No	Solid	Spindle	21	Yes	+ +	+ +	+ +
29	ND	Left	No	Solid	Spindle	4	Yes	+ +	+ +	+
30	12	Left	No	Solid	Spindle	7	Yes	+ +	+ +	+ +
31	13	Right	No	Solid	Epithelioid	34	Yes	+ +	+ +	+ +
32	85	Left	No	Solid	Pleomorphic	12	No	+ +	+ +	+ +

#### Primary AS

**Fig. 2:**
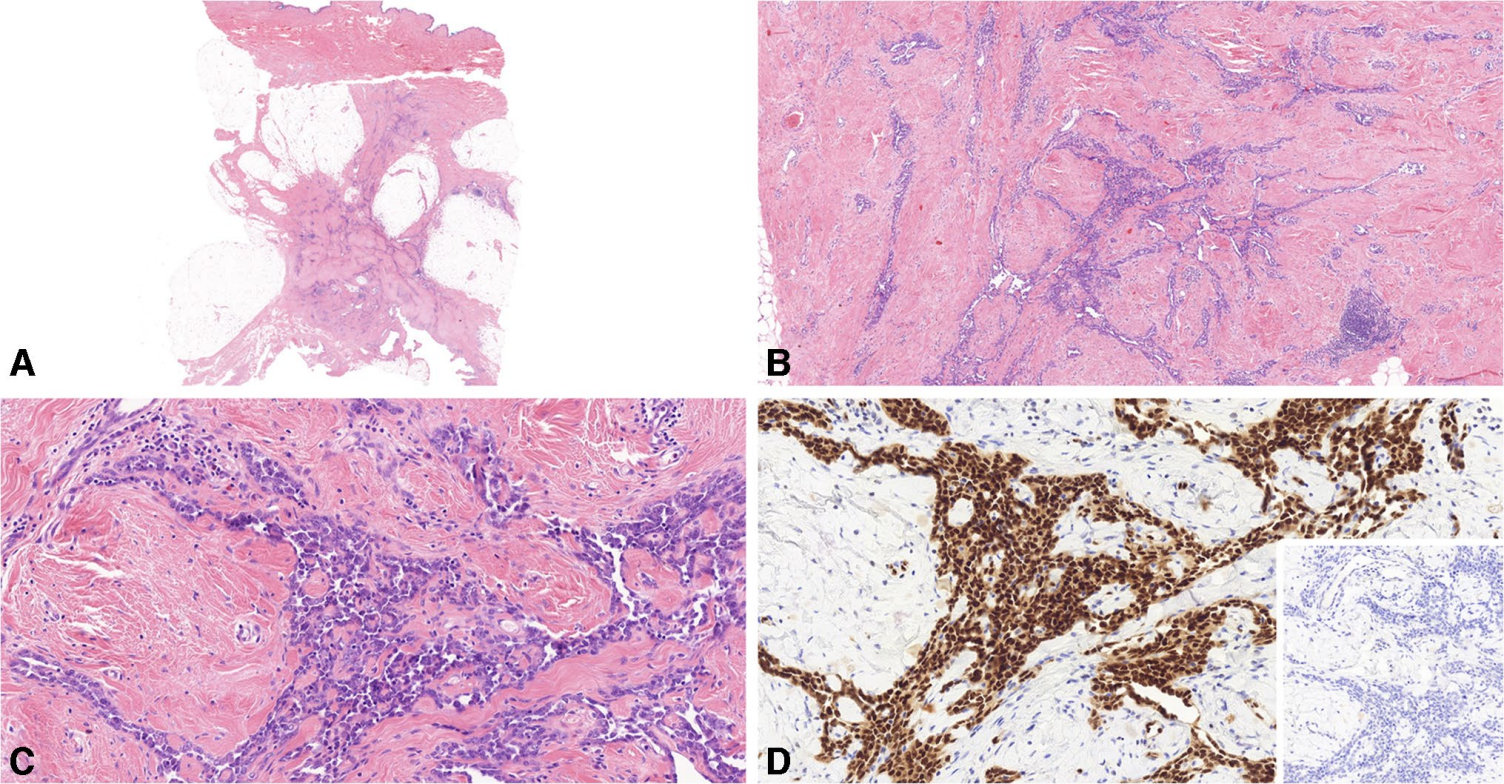
Histological features of primary breast angiosarcoma. **A** On low-power view, a deep-seated, poorly-defined neoplastic tissue is seen in the breast parenchyma (HE × 0.5). **B** Irregularly formed vascular spaces are present in a desmoplastic background (HE × 5). **C** The monomorphous tumor cells exhibit an epithelioid character with prominent nucleoli. In addition, mitotic figures are rare (HE × 20). **D** Immunohistochemical analysis reveals diffuse expression of ERG in tumor cells, while MYC expression is fully absent [insert photo] (both × 20)

The primary AS was situated deep within the parenchyma of the left breast, forming a substantial mass of 21 cm in greatest dimension. The tumor was built up by tiny, epithelioid tumor cells with mild cytological atypia, forming large vascular channels. We saw no necrotic areas, and the mitotic activity was low; hence, the tumor was assigned as a low-grade AS according to the Kuba grading system. Immunohistochemically, the tumor exhibited diffuse positivity for CD31 and ERG, with no discernible expression of MYC and negativity for TRPS1. Representative images are provided in Fig. 2.

### TRPS1 expression

**Fig. 3:**
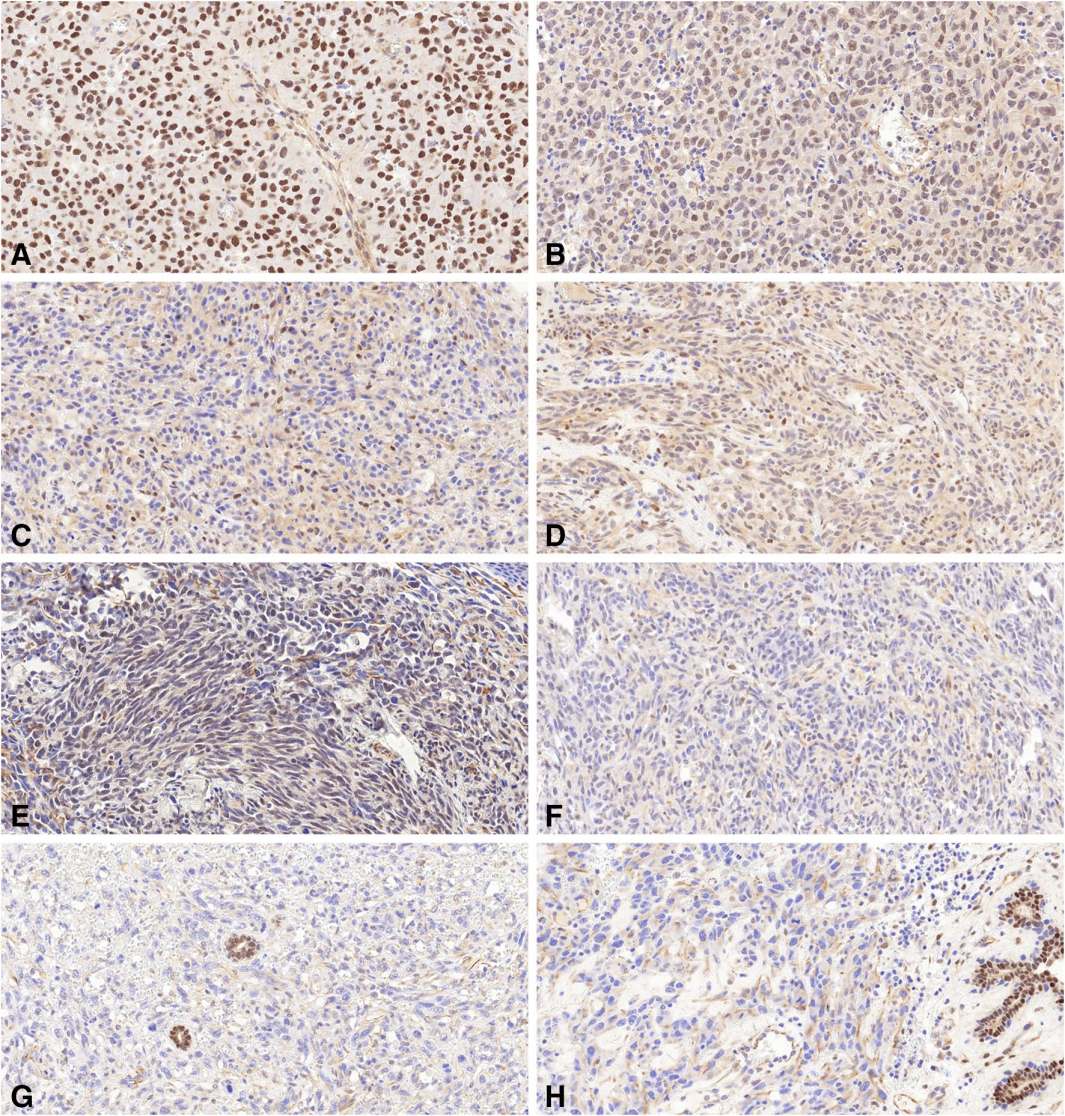
Examples of TRPS1 expression in breast angiosarcomas. **A** In this case, diffuse TRPS1-positivity is evident, characterized by strong nuclear staining. **B** Similar to the previous tumor, there is diffuse TRPS1-positivity, but the reaction’s intensity is weaker. **C** Here, TRPS1-labeling is strong but focal in nature. **D** Tumor cells exhibit weak cytoplasmic background staining, which does not interfere with the identification of nuclear staining. Unanimously, readers deemed these cases positive. **E** In contrast, the intensity of cytoplasmic background staining in this case impedes the validation of any nuclear positivity, rendering TRPS1-positivity doubtful. **F** Tumor cells in this case demonstrate focal TRPS1 expression with weak intensity. While three readers concluded the case as positive, one deemed it negative. Since there was no consensus, TRPS1-positivity remained uncertain. **G**, **H** The last two tumors show no TRPS1 expression. Residual normal ducts of the breast serve as positive internal controls for the reaction (all × 20)

The TRPS1 IHC evaluation results, as summarized in Table 3, revealed unanimous agreement among the readers for 14 cases classified as negative and 12 cases classified as positive. Notably, there was no consensus on the classification of nine tumors, with all of them scored as positive by reader 2 and one or two additional readers. Noteworthy discrepancies were observed in individual cases, such as case 26, where all readers deemed the tumor positive, yet it exhibited no TRPS1 expression upon relapse. Conversely, in case 27, while the primary tumor showed no TRPS1 labeling, its metastasis to the contralateral breast displayed full positivity for TRPS1. Remarkably, the calculated *kappa* value of 0.76 indicates a substantial level of agreement among the readers. According to these findings, at least 34.3% of the AS cases exhibited TRPS1 positivity. Furthermore, among the positive cases, solid and vasoformative architectures were observed in nine and three tumors, respectively. Statistical analysis revealed no significant differences between TRPS1 expression and the dominant growth pattern (*p* = 0.49). Concerning the cell types of the TRPS1 positive cases, six AS cases were composed of pleomorphic cells, three of epithelioid, and three of spindle cells. Additionally, there was no significant difference between the dominant tumor cell morphology and the TRPS1 labeling (*p* = 0.84). Representative images illustrating these results are presented in Fig. 3.

**Table 3:** Evaluation of the TRPS1 immunohistochemistry among the readers. L indicates left; *R*, right; *P*, primary tumor; *Re*, relapse; *M*, metastasis

Patient ID	Reader 1	Reader 2	Reader 3	Reader 4	Consensus
1	Negative (0%)	Negative (0%)	Negative (0%)	Negative (0%)	Yes, negative
2	Negative (0%)	Negative (0%)	Negative (5%)	Negative (0%)	Yes, negative
3	Negative (0%)	Negative (0%)	Negative (5%)	Negative (0%)	Yes, negative
4	Negative (0%)	Negative (0%)	Negative (0%)	Negative (0%)	Yes, negative
5	Negative (0%)	Negative (0%)	Negative (0%)	Negative (0%)	Yes, negative
6	Negative (0%)	Positive (50%)	Positive (60%)	Positive (50%)	No
7	Positive (90%)	Positive (90%)	Positive (80%)	Positive (80%)	Yes, positive
8	Negative (0%)	Negative (0%)	Negative (0%)	Negative (0%)	Yes, negative
9	Negative (0%)	Positive (30%)	Positive (20%)	Negative (5%)	No
10	Positive (10%)	Positive (20%)	Negative (5%)	Negative (5%)	No
11	Positive (50%)	Positive (70%)	Positive (70%)	Positive (100%)	Yes, positive
12	Negative (0%)	Positive (50%)	Positive (20%)	Positive (30%)	No
13	Positive (40%)	Positive (30%)	Negative (5%)	Positive (70%)	No
14	Positive (50%)	Positive (40%)	Positive (50%)	Positive (90%)	Yes, positive
15	Positive (30%)	Positive (60%)	Positive (80%)	Positive (70%)	Yes, positive
16	Negative (0%)	Negative (0%)	Negative (0%)	Negative (0%)	Yes, negative
17	Positive (10%)	Positive (50%)	Negative (5%)	Positive (30%)	No
18	Positive (90%)	Positive (90%)	Positive (80%)	Positive (100%)	Yes, positive
19	Negative (0%)	Positive (20%)	Positive (30%)	Positive (10%)	No
20	Positive (10%)	Positive (25%)	Negative (5%)	Positive (15%)	No
21	Positive (10%)	Positive (30%)	Positive (30%)	Positive (90%)	Yes, positive
22	Negative (0%)	Negative (0%)	Negative (0%)	Negative (0%)	Yes, negative
23	Negative (0%)	Positive (15%)	Negative (1%)	Positive (20%)	No
24	Positive (20%)	Positive (75%)	Positive (40%)	Positive (70%)	Yes, positive
25, L	Negative (0%)	Negative (0%)	Negative (0%)	Negative (0%)	Yes, negative
25, R	Negative (0%)	Negative (0%)	Negative (0%)	Negative (0%)	Yes, negative
26, P	Positive (30%)	Positive (40%)	Positive (20%)	Positive (70%)	Yes, positive
26, Re	Negative (0%)	Negative (0%)	Negative (0%)	Negative (0%)	Yes, negative
27, P	Negative (0%)	Negative (0%)	Negative (0%)	Negative (5%)	Yes, negative
27, M	Positive (20%)	Positive (40%)	Positive (10%)	Positive (30%)	Yes, positive
28	Positive (30%)	Positive (40%)	Positive (20%)	Positive (60%)	Yes, positive
29	Negative (0%)	Negative (0%)	Negative (0%)	Negative (0%)	Yes, negative
30	Positive (20%)	Positive (40%)	Positive (100%)	Positive (80%)	Yes, positive
31	Positive (100%)	Positive (90%)	Positive (100%)	Positive (100%)	Yes, positive
32	Negative (5%)	Negative (5%)	Negative (1%)	Negative (0%)	Yes, negative

## Discussion

The two manifestations of breast angiosarcoma exhibit distinctions in etiology, epidemiology, pathology, and genetic characteristics. Primary AS originates deep within the breast parenchyma and may extend to involve the skin [4]. Notably, primary AS predominantly affects younger women, with a median age of approximately 40 years [4]. It is worth mentioning that within our cohort, an exceptional case involved a 33-year-old female patient diagnosed with Milroy syndrome, which is a genetic disorder associated with chronic lymphedema in various tissues [18]. Importantly, primary AS shows no correlation with previous breast surgery or radiation therapy. In contrast, secondary AS of the breast is a well-established rare and delayed complication arising from breast cancer treatment [19]. RAAS is related to the DNA damage and genomic instability induced by the ionizing energy [20]. Some studies suggest a dose-dependent risk for RAAS [18]. Given that breast cancer typically afflicts the elderly, the peak incidence of RAAS occurs between 60 and 70 years, typically emerging 6 to 10 years after breast irradiation [21–23]. In our studied cohort, we observed a similar age distribution, with a median age of 63 years. On average, 8 years elapsed after the radiotherapy for primary breast cancer before the development of RAAS.

Secondary AS of the breast can both clinically and pathologically present as something else. In rare instances, the histological appearance of the lesion can be deceptively benign, exhibiting a resemblance to hemangiomas. Notably, the late Professor Juan Rosai considered the presence of a capillary lobule, which can be part of the morphology of postirradiation AS, as practically diagnostic of a benign vascular proliferation [24]. On the other end of the spectrum, epithelioid cells of AS may mimic recurrent carcinomas, and their occasional positivity with keratin antibodies may further contribute to the possibility of a diagnostic pitfall; such cases can present both clinically and under the microscope as recurrent carcinomas [25, 26]. To avoid this diagnostic trap, it is advisable to incorporate certain immunohistochemical markers specific to breast carcinoma in the diagnostic process. GATA3 and SOX10 are commonly employed markers reliably indicating breast origin; however, they do have limitations [27, 28]. It is noteworthy that these markers are not entirely breast-specific, as GATA3 can be expressed in certain renal neoplasms, neuroendocrine neoplasms, and urothelial carcinomas [29, 30]. Similarly, SOX10 staining is characteristic of melanocytic tumors and soft tissue neoplasms with neural crest origin [31, 32]. In the context of breast carcinomas, GATA3 is predominantly expressed in hormone receptor-positive tumors (luminal A and luminal B) and HER2-positive carcinomas [33], while SOX10 serves as a hallmark stain for triple-negative (basal-like) carcinomas [26].

A promising alternative is TRPS1, which has garnered attention due to its widespread expression, regardless of hormone receptor status, making it a versatile marker in breast carcinoma diagnostics [10, 14]. Our group reinforced the value of TRPS1 expression in breast carcinomas with a triple-negative phenotype [15]. On the other hand, it is crucial to acknowledge that there are limited data available regarding TRPS1 positivity in other tumor types. In a study conducted by Ai et al., which investigated TRPS1 expression in 1234 malignant tumors, non-mammary TRPS1 labeling was exceptionally rare [10]. It is important to note that this study utilized tissue microarray blocks and specifically focused on evaluating breast-specific immunohistochemical markers; hence, various carcinomas and melanomas were the only included entities in the analysis [10].

In a very recently published paper by Bachert and colleagues, TRPS1 expression was reported at a rate of approximately 31% and 27% in prostate and urothelial carcinomas, respectively [34]. Notably, the study revealed a surprising finding: a significant percentage of lymphatic and distant metastases in prostate adenocarcinoma retained TRPS1 positivity [34]. The expression of TRPS1 in urothelial and prostate carcinoma remains inadequately elucidated. While the PA5-84874 clone has been predominantly utilized in most studies, a singular investigation employed an alternative conventional antibody, namely the EPR16171 clone [35]. Future research endeavors could be directed towards comparing the specificity and sensitivity of these clones specifically in breast carcinoma and exploring their expression profiles across various tumor types.

Additionally, Wang and his colleagues explored TRPS1 immunohistochemistry in mesenchymal tumors [13]. Concerning mammary sarcomas, they observed high TRPS1 expression in malignant phyllodes tumors, while no expression was detected in other breast mesenchymal tumors, including AS [13]. Furthermore, considerable TRPS1 labeling was noted in extramammary osteosarcoma and chondrosarcoma [13]. Our observations stand in contrast to these findings, as 34.3% of our angiosarcomas exhibited unequivocal TRPS1 labeling, while in nine cases (25.7%), TRPS1 expression was deemed uncertain. An intriguing case in our study involved an AS with a corresponding local relapse, where unexpectedly, the primary tumor was identified as positive for TRPS1, while all readers considered the relapse negative. Another noteworthy instance was a case featuring matching primary AS and distant metastasis, where we observed a reversed staining pattern, namely TRPS1 negativity for the primary tumor and TRPS1 positivity for the distant metastasis. Based on the latter case, caution is warranted when determining breast carcinoma origin based solely on TRPS1 positivity. Regarding other mesenchymal tumors, Cloutier and colleagues observed heightened TRPS1 expression in both primary and metastatic synovial sarcomas [12]. Their conclusion pointed towards the influence of the SS18-SSX oncoprotein on TRPS1 expression in these tumors [12].

In our analysis, the readers achieved substantial agreement in evaluating TRPS1 labeling. Positive immunohistochemical reactions were considered when at least 10% of tumor cells displayed labeling at any staining intensity. This threshold had been previously established by our group in testing TRPS1 expression in breast carcinomas [15], aligning with the approach used by the group of Wang and colleagues [13]. Notably, we categorized a case as either positive or negative for TRPS1 only when there was unanimous agreement among the readers. Consequently, 40%, 34.3%, and 25.7% of AS cases were classified as negative, positive, and doubtful, respectively. RAAS usually exhibit distinctive morphological features (vessel-forming tumor) and occur in a specific clinical context (developing years after breast cancer treatment) [21]. However, it is crucial to emphasize that in cases where clinical data are unavailable or uncommunicated, and the vessel formation is not characteristic, a positive or doubtful TRPS1 immunohistochemical reaction may lead to an erroneous diagnostic conclusion. In our experience, 28 AS cases had a dominant solid morphology, and among these, for TRPS1, nine were labeled as positive and seven as doubtful by the readers. These findings suggest that in 47% of AS cases, there may be a potential for misdiagnosis as dedifferentiated breast carcinoma. Particularly in cases with limited tissue samples, such as core biopsies, reliance on TRPS1 expression alone may lead to an erroneous diagnostic trajectory.

Considering the current hypothesis that angiosarcomas originate from blood vessel and lymphatic endothelial cells, the expression of endothelial markers such as FLI1, ERG, CD31, and CD34 becomes pivotal [34]. Both CD31 and ERG strongly stain both blood and lymphatic vessel endothelial cells, making the combined application of these antibodies particularly beneficial in confirming the endothelial nature of the tumor [37, 38]. In our set, CD31 and ERG stained virtually 100% of the tumor cells. Actually, the lack of the endothelial marker expression led us to exclude the two wrongly diagnosed cases.

The genetic profiles of primary AS and RAAS exhibit notable distinctions. Primary AS often harbors activating mutations of the MAPK pathway, coupled with the loss of the *TP53* gene [39]. In contrast, a hallmark genetic alteration in RAAS involves the amplification of the *MYC* gene [40], typically exceeding 100 copies, located at the 8q24.21 locus [41]. The identification of this alteration can be achieved through fluorescent in situ hybridization, or alternatively, by detecting the overexpression of the MYC protein using immunohistochemistry [40]. Co-amplification of the *FLT4* gene has been reported in 25% of RAAS cases, suggesting a potential secondary hit in the pathogenesis of RAAS [42]. Additionally, the downregulation of miR-34c, a regulatory microRNA of *MYC*, has been observed in RAAS [36]. Although mutations in the DNA repair genes *BRCA1* and *BRCA2* have been detected in RAAS, their precise impact on tumorigenesis remains a subject of debate [43]. In our experience, MYC was expressed in a diffuse and focal fashion in 29 and six RAAS cases, respectively. Our single primary AS case was completely negative for MYC expression. The inclusion of a single primary AS case and its negativity for TRPS1 does not allow firm conclusions on the expression of the marker in this entity.

In summary, our study establishes that a notable proportion of breast AS expresses TRPS1, challenging the previously asserted high specificity of TRPS1 for breast carcinomas. This discovery broadens the spectrum of entities expressing TRPS1, impacting the differential diagnosis. A potential pitfall emerges with high-grade breast tumors exhibiting a solid growing pattern, as TRPS1 alone may not effectively distinguish between undifferentiated breast carcinoma and AS. While we recognize TRPS1 as a valuable marker, we advocate for its combined use with either SOX10 or GATA3 to achieve the highest diagnostic accuracy. Notably, in our investigation, both CD31 and ERG proved equally effective as markers in confirming the endothelial origin of the tumors. Also, MYC protein was expressed in all RAAS cases indicating the pathognomonic genetic alteration of these tumors. Our study prompts the question of the underlying reasons and clinical relevance for TRPS1 expression in these tumors. Future endeavors include planned comprehensive molecular investigations to shed light on this intriguing aspect.

## Supplementary Information

Below is the link to the electronic supplementary material.

Suppl. Fig. 1 Examples of macroscopic appearances of radiation-associated angiosarcoma (RAAS). a RAAS originates from irradiated skin, manifesting as erythematous plaques and nodules characterized by irregular shapes and sizes. b, c In more advanced stages, RAAS develops into voluminous masses on the skin's surface, exhibiting extensive invasion into the breast parenchyma (PNG 397 KB)

## Data Availability

All data generated or analyzed during this study are included in this article. Further inquiries can be directed to the corresponding author.

## Abbreviations

AS: Angiosarcoma
RAAS: Radiation-induced angiosarcoma
AVL: Atypical vascular lesion
TRPS1: Trichorhinophalangeal syndrome protein 1
IHC: Immunohistochemistry
