# Supplementary figures and images for: TRPS1 expression in breast angiosarcoma
Source: Virchows Arch. 2024 Jun 20;486(3):479–90. doi: 10.1007/s00428-024-03852-2 (PMC11950113; doi:10.1007/s00428-024-03852-2)

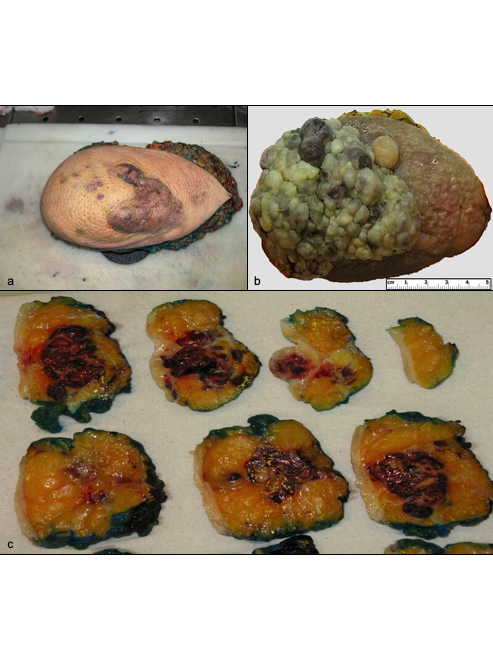

Supplement: Supplementary file 1 — Examples of macroscopic appearances of radiation-associated angiosarcoma (RAAS). a RAAS originates from irradiated skin, manifesting as erythematous plaques and nodules characterized by irregular shapes and sizes. b, c In more advanced stages, RAAS develops into voluminous masses on the skin's surface, exhibiting extensive invasion into the breast parenchyma (PNG 397 KB) [file 428_2024_3852_MOESM1_ESM.png]
